# CSF drainage strategy and complication management of hydrocephalus

**DOI:** 10.3389/fneur.2026.1813065

**Published:** 2026-09-28

**Authors:** Chunsheng Xia, Zhixiong Lin, Xiangpin Wei, Hao Xu

**Affiliations:** 1 Department of Neurosurgery, The First Affiliated Hospital of USTC, Division of Life Sciences and Medicine, University of Science and Technology of China, Hefei, Anhui, China; 2 Department of Neurosurgery, Sanbo Brain Hospital, Capital Medical University, Beijing, China

**Keywords:** cerebrospinal fluid drainage, complication management, external ventricular drainage, hydrocephalus, ventriculoperitoneal shunt

## Abstract

**Background and objectives:**

Conventional CSF drainage strategies—using external ventricular drainage (EVD) for acute hydrocephalus and permanent shunts (ventriculoperitoneal [VP], ventriculoatrial [VA], lumboperitoneal [LP]) for chronic hydrocephalus-are often inadequate in complex clinical scenarios (e.g., patients with intracranial hemorrhage, active infection, or unstable conditions who cannot undergo permanent shunt surgery). This creates an urgent need to comprehensively review drainage modalities to address the core challenge of reducing surgical frequency while effectively controlling intracranial pressure (ICP) and avoiding complications.

**Methods:**

This review systematically examines seven major CSF drainage methods: EVD, lumbar drainage (LD), Ommaya reservoir EVD, long-tunneled EVD (LTEVD), VP shunt, VA shunt, and LP shunt. It details each method’s indications, contraindications, advantages, disadvantages, and typical application duration based on clinical evidence and literature. Additionally, the review covers key aspects of external drainage management and targeted strategies for handling common complications.

**Expected outcomes:**

This review aims to provide neurosurgeons with a practical, evidence-based reference for selecting optimal CSF drainage strategies. It also proposes a stepwise clinical protocol to guide decision-making—from initial intervention for acute hydrocephalus to transition to long-term or permanent drainage solutions—with the goal of balancing reduced surgery numbers, effective ICP control, and complication prevention to improve patient prognosis.

**Discussion:**

A practically feasible protocol with preliminary clinical evidence is proposed: initiate EVD for acute hydrocephalus; for shunt-dependent patients, use LD for communicating hydrocephalus or Ommaya reservoir EVD for obstructive hydrocephalus; if permanent shunts remain unfeasible after re-evaluation, switch to LTEVD; and finally transition to appropriate permanent shunts when conditions permit. This protocol optimizes drainage strategies, addresses gaps in traditional approaches, and supports neurosurgeons in personalized treatment selection.

## Background

Hydrocephalus, defined by abnormal cerebrospinal fluid (CSF) accumulation in the ventricular system, requires surgery to prevent neurological decline. Its classic treatment involves: external ventricular drainage (EVD) for acute cases (with subsequent tube removal); and permanent shunts (ventriculoperitoneal [VP], ventriculoatrial [VA], or lumboperitoneal [LP]) for chronic cases. However, this classic strategy faces practical clinical challenges, and no solution with outstanding advantages exists (1–3).

Clinically, some hydrocephalus patients cannot undergo permanent shunt surgery due to intracranial hemorrhage, active infection, or unstable conditions (4). These patients need temporary/alternative therapies to relieve symptoms while reducing surgical frequency—the core challenge is minimizing surgeries while controlling intracranial pressure (ICP) and avoiding complications.

Beyond traditional EVD, multiple modified approaches exist. For example, Ommaya reservoir-based EVD (4, 5), usually indwelling for 7–10 days, maybe employed over extended durations, even for several weeks (6–10). Though scalp puncture site changes allow long-term indwelling, it brings care difficulties (e.g., post-puncture reservoir fixation, easy needle displacement/detachment), increased patient discomfort, higher costs, and elevated infection risks (even secondary surgery for drainage site replacement) from repeated punctures. In recent years, long-tunneled external ventricular drainage (LTEVD) and modified ventriculoperitoneal shunts (mVPS, externalizing the VP shunt’s abdominal end) have been reported, especially for patients with hydrocephalus combined with intracranial infection and/or high CSF protein (11–14). Lumbar drainage (LD) can also be transitional but fails to solve long-term indwelling tube management issues.

This review focuses on major CSF drainage modalities (EVD, LD, Ommaya EVD, LTEVD, VP, VA, LP), detailing their indications, pros, cons, and typical application durations based on references (Figure 1 and Table 1), aiming to provide practical support for neurosurgeons in drainage strategy selection.

**Figure 1:**
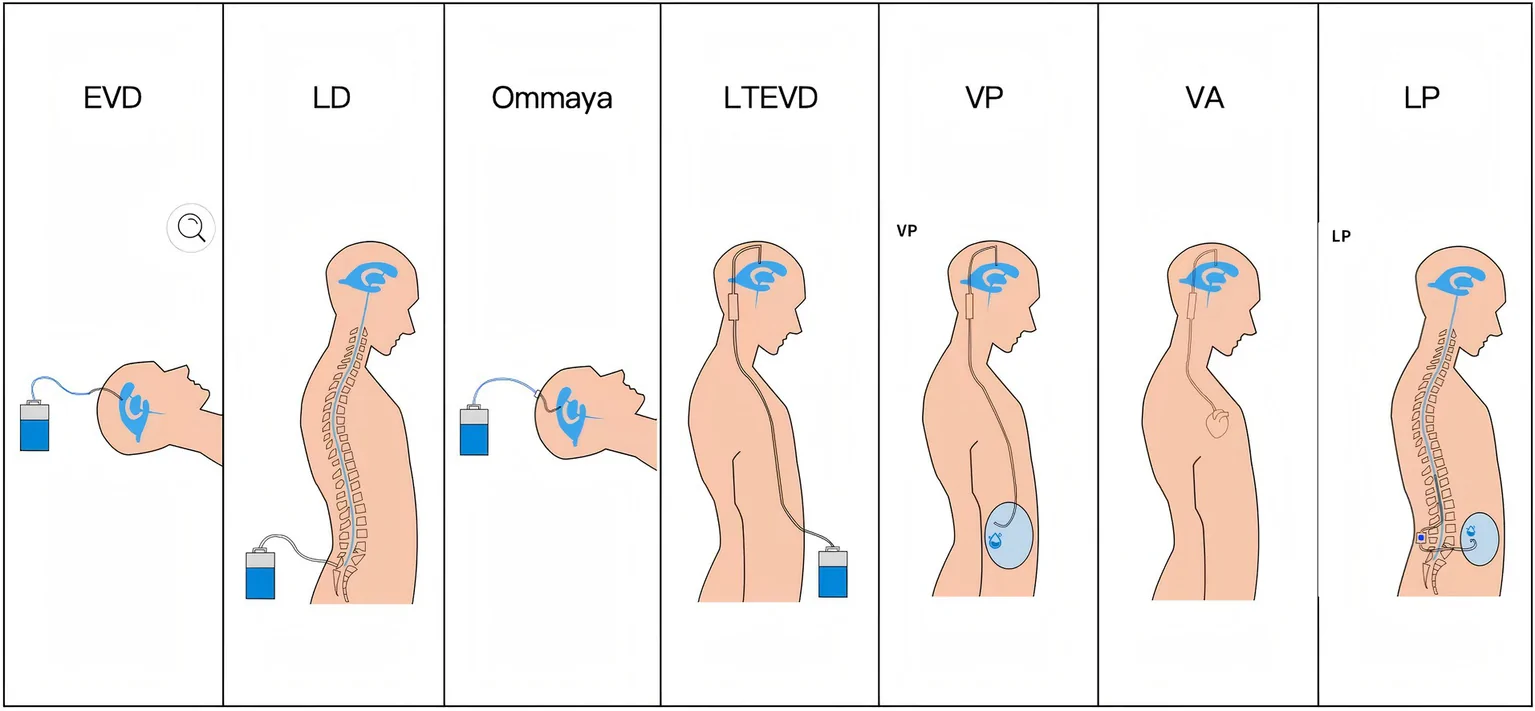
Seven different types of cerebrospinal fluid drainage strategies.

**Table 1:** Indications, advantages, disadvantages and typical application durations for different CSF drainage method.

Method	Indications	Advantages	Disadvantages	Typical duration
EVD	Acute hydrocephalus, ICP monitoring, intraventricular hemorrhage	Real-time ICP monitoring, rapid drainage, drug administration	High infection risk, limited mobility, over/under-drainage risk	7–10 days (max 14 days)
LD	Subarachnoid hemorrhage (Fisher 3–4), CSF leaks, meningitis	Less invasive, easy to perform, lower parenchymal injury risk	No ICP monitoring, risk of herniation with over-drainage	Short-term (max 14 days)
Ommaya reservoir EVD	Long-term CSF access, intraventricular hemorrhage, drug administration	Reduced infection risk, allows mobility, repeated sampling	Surgical implantation needed, risk of occlusion/infection	Weeks
LTEVD	Prolonged drainage needs, intracranial infections with hydrocephalus	Longer indwelling time, lower infection rate allows mobilty, effective long-term	Complex insertion, higher displacement risk, thin catheter prone to occlusion	Weeks to months or even longer
VP shunt	Chronic hydrocephalus, normal pressure hydrocephalus	Classic surgical approach	Obstruction, infection, over-drainage; may need revisions	Permanent
LP shunt	Communicating hydrocephalus, normal pressure hydrocephalus	Less invasive, avoids ventricular puncture	Higher obstruction risk, not for obstructive cases, spinal headache risk	Permanent
VA shunt	Hydrocephalus with abdominal pathology (e.g., peritoneal inflammation)	Applicable to patients with unfavorable abdominal status	Risks of atrial-related complications such as thrombosis, arrhythmia, infective endocarditis, etc.	Permanent

Literature retrieval was performed in four mainstream databases (PubMed, Embase, Web of Science, and Cochrane Library) from database establishment to April 2026. Relevant keywords concerning hydrocephalus and transitional cerebrospinal fluid drainage strategies (LTEVD and Ommaya reservoir drainage) were used for searching, with no language restrictions. Duplicates were removed, and eligible studies were independently screened by two authors based on predefined inclusion and exclusion criteria. Clinical studies, systematic reviews, and authoritative guidelines focused on transitional CSF drainage were included, whereas irrelevant studies, animal experiments, case reports, and conference abstracts were excluded. This work complements the missing content of traditional reviews on staged transitional drainage management, forming a targeted clinical reference for the sequential treatment of complex hydrocephalus.

## CSF drainage methods

### External ventricular drainage (EVD)

External Ventricular Drainage (EVD) is primarily indicated for acute symptomatic hydrocephalus, intracerebral hemorrhage requiring cerebrospinal fluid (CSF) release, acute brain injury needing intraventricular intracranial pressure (ICP) monitoring, neurotumor perioperative management, normal pressure hydrocephalus assessment, and ventriculitis or meningitis treatment via intraventricular medication administration. Its relative contraindications include coagulopathy and skin infection at the puncture site, with no absolute contraindications. The key advantages of EVD are real-time ICP monitoring, rapid CSF diversion, and the ability to administer intraventricular drugs, with adjustability based on patient conditions. However, it has high infection risk (0% ~ 32%) with prolonged use, risks of over-drainage or under-drainage, and limits patient mobility. In addition, conventional EVD requires strict bed rest, which limits patient mobility, hinders early neurological rehabilitation, and increases the risk of bedridden complications. Typically, EVD is used for 7–10 days, not exceeding 2 weeks; if longer drainage is needed, reinsertion at another site is recommended (6, 7, 15).

### Lumbar drainage

Lumbar Drainage (LD) is indicated for selected Fisher 3–4 grade subarachnoid hemorrhage, partial ventricular hemorrhage, central nervous system infections requiring antibiotic treatment, auxiliary therapy for CSF leaks, and neurotumor perioperative preparation to relax brain tissue. Its absolute contraindication is cerebral herniation, while relative contraindications include severe intracranial hypertension, lumbar spine deformities, severe systemic infection, high cervical spinal cord space-occupying lesions, incomplete CSF circulation obstruction, and uncooperative patients. LD is less invasive than EVD, with a lower risk of parenchymal injury and easier operation (16). However, it cannot monitor ICP, carries a risk of over-drainage leading to intracranial hemorrhage or herniation, and is contraindicated in severe intracranial hypertension. It is generally used for a short period; in bacterial meningitis, it can be used continuously based on the patient’s condition, and for refractory CSF leaks, it is used before surgical repair after failed conservative treatment. Some studies and Meta analysis suggest that LD can effectively reduce the incidence of shunt-dependent hydrocephalus in patients with subarachnoid hemorrhage (17). However, it is particularly important to note that LD is dangerous for obstructive hydrocephalus (18, 19).

### Ommaya reservoir EVD

The Ommaya reservoir is indicated for repeated access to the intrathecal space, combined use with external ventricular drain (EVD) in the management of intraventricular hemorrhage, scenarios requiring long-term cerebrospinal fluid (CSF) drainage, and intrathecal administration of chemotherapeutic or antimicrobial agents. Its contraindications are comparable to those for EVD placement, including coagulopathy and local site infection, and caution is advised in patients with uncontrolled systemic infection. Compared with prolonged EVD placement, Ommaya reservoir may lower the risk of infection, permit patient mobilization, and facilitate repeated CSF sampling and intrathecal drug delivery. Nevertheless, surgical implantation is required, with associated risks of reservoir-related infection and catheter occlusion; furthermore, several studies have demonstrated no significant improvement in functional outcomes relative to EVD alone. The device can serve for long-term use over weeks to months, according to individual clinical requirements. Zhu et al. (20] described cases in which Ommaya reservoirs were maintained for more than 2 months before conversion to ventriculoperitoneal (VP) shunts, without evidence of recurrent infection. Singh (21] suggested that conversion to Ommaya reservoir-mediated drainage may represent a reasonable alternative when complications arise with an existing EVD.

### Long-tunneled external ventricular drainage (LTEVD)

Long-Tunneled External Ventricular Drainage (LTEVD) is indicated for prolonged CSF drainage needs, intracranial infections with hydrocephalus, and cases where standard EVD duration is insufficient. Long-tunneled EVD catheters were tunneled subcutaneously for approximately 50–60 cm using malleable shunt passers and externalized on the lower chest, upper abdomen or even lower abdomen. LTEVD has a significantly longer indwelling time than standard EVD, a lower daily infection rate, and a reduced risk of CSF leakage (22] However, its insertion is more complex, with a higher risk of catheter displacement, and the relatively thin catheter may be prone to occlusion (23). The median duration of use is 23 days (range 14–35 days), and it can be extended to several months in selected cases (24, 25). For patients who cannot meet the criteria for internal cerebrospinal fluid drainage for a long time, this is almost the most advantageous method.

### Ventriculoperitoneal shunt

Ventriculoperitoneal (VP) Shunt is indicated for chronic hydrocephalus, normal pressure hydrocephalus, and post-hemorrhagic hydrocephalus requiring permanent CSF diversion (10). Its contraindications include active intracranial or abdominal infection, uncorrected coagulopathy, and severe abdominal pathology preventing peritoneal catheter placement (26). It provides permanent CSF diversion, allows normal patient mobility, and is effective for long-term management (11). However, it carries risks of shunt obstruction, infection, and over-drainage, may require revision surgery, and is associated with long-term complications (27). It is used permanently, with a variable lifespan depending on patient factors and device performance. VP shunting is the most common and widely used method. However, its inherent complications are always present, requiring some other supplementary approaches to address specific special situations.

6. Lumboperitoneal (LP) Shunt

Lumboperitoneal (LP) Shunt is indicated for communicating hydrocephalus, normal pressure hydrocephalus, pseudotumor cerebri, and cases where ventricular access is difficult (10). Its contraindications include obstructive hydrocephalus, lumbar spine abnormalities, infection at the insertion site, and coagulopathy. It is less invasive than VP shunt, avoids ventricular puncture, and has a lower risk of brain parenchymal injury. However, it has a higher risk of shunt obstruction due to the lumbar catheter position, cannot be used in obstructive hydrocephalus, and may cause spinal headache (28, 29). LP is a surgical procedure with minimal trauma and is most easily accepted by patients, especially suitable for normal pressure hydrocephalus. For normal pressure hydrocephalus, comprehensive preoperative assessment is required, and the optimal drainage strategy should be selected in accordance with clinical guidelines.

### Ventriculoatrial shunt

Ventriculoatrial (VA) Shunt is indicated for hydrocephalus in patients with abdominal pathology precluding VP shunt, such as chronic peritoneal inflammation or infection. Its contraindications include cardiac anomalies, severe cardiovascular disease, coagulopathy, and systemic infection (30). It serves as an alternative when peritoneal access is not feasible and effectively diverts CSF into the systemic circulation (31). However, it has a higher risk of systemic complications (e.g., endocarditis, thromboembolism), requires vascular access, and is more complex to insert. It is used permanently but may have higher complication rates requiring earlier revision compared to VP shunts (32).

## Management of external ventricular drainage

Unlike internal cerebrospinal fluid (CSF) drainage, the most crucial aspect of external drainage lies in the management of the drainage tube. A great many complications arise from inadequate management of the drainage tube (33). For example, cerebrospinal fluid leak at percutaneous exit of ventricular catheter would greatly increase the risk of infection (34). The wound dressing should be kept clean and dry, with enhanced disinfection of the skin around the drainage tube exit. It is crucial to check if the drainage tube length is sufficient, whether there is kinking or compression, and if the tube position is appropriate. Observe whether the fluid level in the tube fluctuates with respiration or pulse-an absence of fluctuation in the early stage of drainage mostly indicates tube occlusion. Gentle squeezing of the tube can be performed to ensure patency, and reinsertion is necessary if obstruction persists. Record the volume and color of drained CSF per unit time. The patient’s head movement should be appropriately restricted; for agitated or uncooperative patients, protective restraints and sedative-analgesic therapy are recommended (1, 35, 36).

## Drainage volume and patient position management

External drainage tubes do not have anti-siphon devices, and the drainage volume must be controlled entirely by adjusting the position of the drainage bottle. The ventricular drainage bottle should be hung at the bedside, with the highest point of the drainage tube positioned 10–15 cm above the lateral ventricle level (for supine patients: at the horizontal plane of the midpoint between the external canthus and external auditory meatus; for lateral decubitus patients: at the mid-sagittal plane).

The daily CSF drainage volume should generally not exceed 500 mL (normal CSF secretion is 400–500 mL/day), with most cases controlled at approximately 200 mL per day and an average drainage rate of <15–20 mL/h. Exceeding this rate in lumbar drainage (LD) may lead to intracranial hemorrhage or even cerebral herniation.

Since many patients need to carry drainage devices for a long time, restricting their daily activities will affect the recovery of their neurological functions and cause complications such as pulmonary infection and venous thrombosis. Therefore, we encourage patients to carry drainage tubes for daily activities, but it is necessary to ensure that the drainage tubes are firmly fixed. The drainage tube must be clamped before moving the patient or changing their position to prevent abnormal changes in drainage volume, retrograde flow, or tube dislodgement. Upon returning to the ward, the tube should be reopened promptly (1).

## Management of complications

### Hemorrhage

Catheter placement may cause puncture tract hemorrhage, with related causes including vascular abnormalities, excessively large catheter diameter, use of antiplatelet or anticoagulant drugs, elevated international normalized ratio (INR), and excessive CSF drainage. In particular, excessive drainage volume and overly rapid drainage speed may also lead to delayed bleeding in the puncture tract (2). Therefore, the management of drainage volume is crucial for preventing bleeding.

For puncture tract hemorrhage, the main approach is to keep the drainage tube unobstructed, and urokinase can be injected if necessary. In our clinic practice, massive bleeding in the puncture tract is rare, but once it occurs, craniotomy may be required to remove the hematoma. If there is a permanent shunt, accumulated blood will quickly block the drainage tube. In such cases, the shunt should be closed, and EVD or LD could be performed immediately. There is currently no consensus on when to resume the internal drainage device. We believe that after the drainage fluid is completely clear, the shunt can be restart. In many cases, the drainage tube can be reused.

### Infection

Once a VP shunt infected, antibiotic therapy and continuous drainage of infected CSF are typically required. At this point, implantation of an EVD or LD may be necessary, though both EVD and LD carry inherent risks of infection. Key preventive strategies encompass the implementation of rigorous aseptic protocols, the avoidance of tube leakage and retrograde flow, the prevention of contact between the external catheter opening and the fluid within the cerebrospinal fluid collection bottle, as well as the clamping of the tube during transportation for diagnostic evaluations (37).

Secondary purulent ventriculitis and meningitis represent the most severe complications of CSF external drainage, with intracranial infection rates is 23%. A meta analysis showed 7 potential risk factors (SAH/IVH, concomitant systemic infection, other neurosurgical procedures, change of catheter, bilateral EVDs, CSF leak, and duration of EVD > 7 days) were identified (38).

In cases where infection control is suboptimal, intrathecal antibiotic injection or intraventricular antibiotic infusion can be employed. We commonly utilize LD for antibiotic administration; if this proves ineffective, antibiotic injection via an Ommaya reservoir combined with unobstructed LD drainage may be adopted (39, 40).

### Obstruction

The probability of obstruction in VP shunts and LP shunts is generally considered to be mechanical obstruction. In contrast, EVD and LD have a higher probability of obstruction because they often need to drain hemorrhagic or infected cerebrospinal fluid. Obstruction may result from clot or sediment blockage, or tube position changes (44). Pathological CSF values such as WBC count and persistently elevated protein level serves as a risk factor for early valve obstruction. Late obstruction occurs independent of normal CSF values (41).

For internal drainage, it is crucial to strictly adhere to the indications and ensure the clarity of cerebrospinal fluid to reduce the probability of obstruction, and there is an abundance of research on this aspect. However, for external drainage, more attention needs to be paid to preventing obstruction and displacement (42). Larger-diameter catheters could reduce the risk of tube obstruction; repeatedly squeeze the tube in cases of intraventricular hemorrhage or suspected clot obstruction; administer thrombolytic agents via the tube for large clots.

### Over-drainage

Over-drainage can cause subdural or epidural hematomas, subdural effusions, aneurysm re-rupture, intracranial hypotension, paradoxical herniation, and intracranial pneumatosis. This issue carries risks in both internal and external drainage. For external drainage, a greater risk lies in massive drainage over a short period due to changes in body position; for internal drainage, it is sometimes difficult to monitor the drainage volume, making over-drainage prone to missed diagnosis. Therefore, for VP shunts, it is advisable to set the initial pressure slightly higher, followed by a cautious and gradual reduction of the shunt pressure (43). It is recommended to set drainage volume based on intracranial pressure assessment. For patients with decompressive craniectomy and EVD, elastic bandages can be used to constrain the skull defect area to prevent intracranial hypotension.

Beyond that, complications of cerebrospinal fluid drainage include displacement of the drainage tube, intracranial pneumatosis, epileptic seizures, cerebrospinal fluid leakage, and shunt dysfunction. However, the incidence of these complications is relatively low, and most of them can improve with symptomatic treatment.

## Cerebrospinal fluid drainage protocol

Based on existing literature and clinical experience, we developed a protocol that has achieved Promising clinical outcomes have been noted following pilot adoption in several hospitals.: For acute hydrocephalus, initiate EVD (max 7–10 days) (1, 2]; if not shunt-dependent, remove EVD; if shunt-dependent, use LD for communicating hydrocephalus or Ommaya reservoir EVD for obstructive hydrocephalus. Re-evaluate weeks after LD/Ommaya: if still shunt-dependent and standard shunts unfeasible, use LTEVD (1–6 months or longer); transition to appropriate permanent shunts (VP/VA for obstructive, VP/VA/LP for communicating) when conditions permit (no active infection, normal coagulation, safe catheter placement, suitable CSF state) (Figure 2).

**Figure 2:**
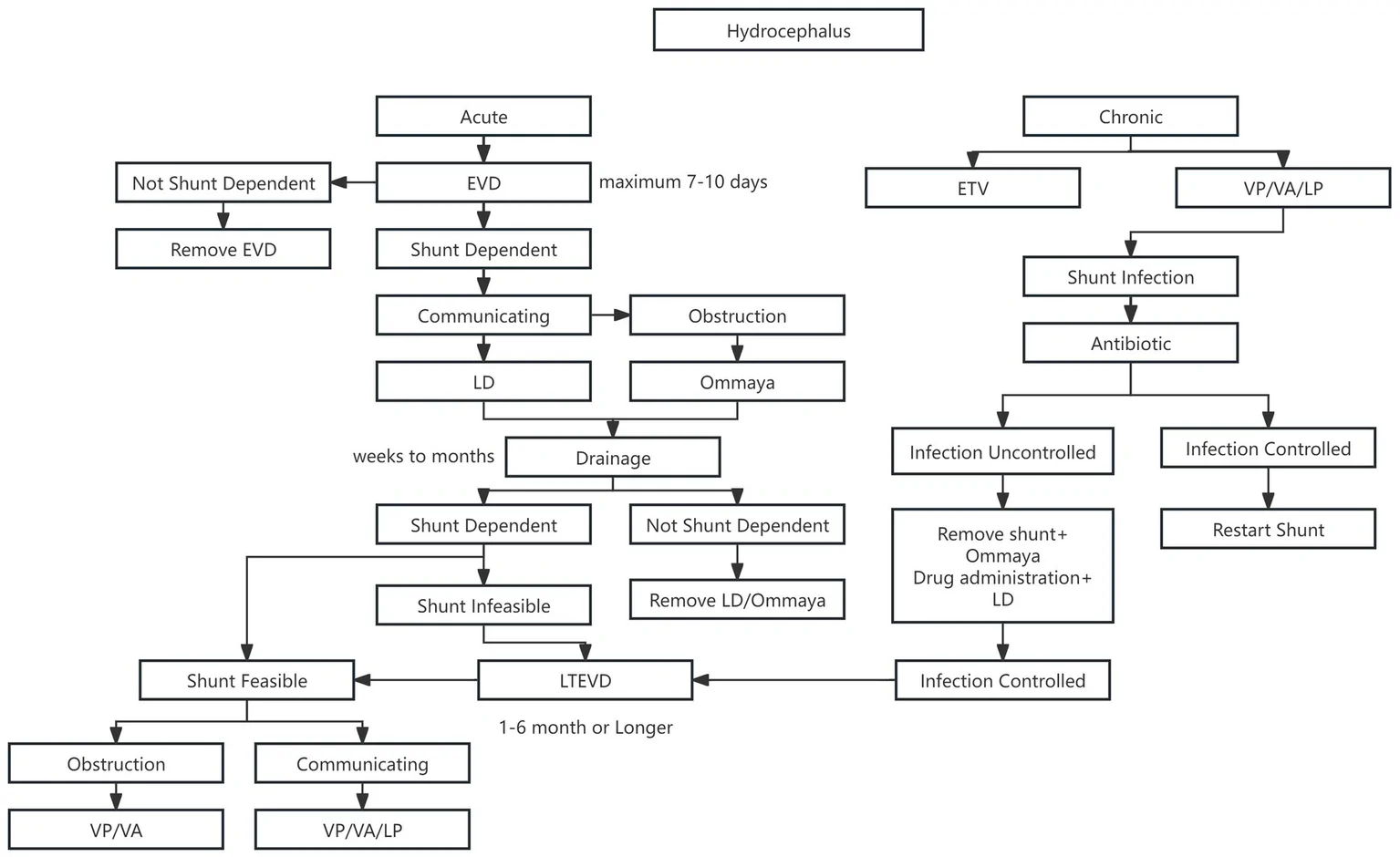
Protocol for surgical method selection and clinical management of acute and chronic hydrocephalus.

Notably, transitional options exist between traditional EVD and permanent shunts: e.g., combining Ommaya reservoir EVD with EVD extends drainage duration and enables drug injection for intracranial infection; LTEVD fills the gap for long-term drainage needs (e.g., post-shunt removal due to infection) (Figure 3).

**Figure 3:**
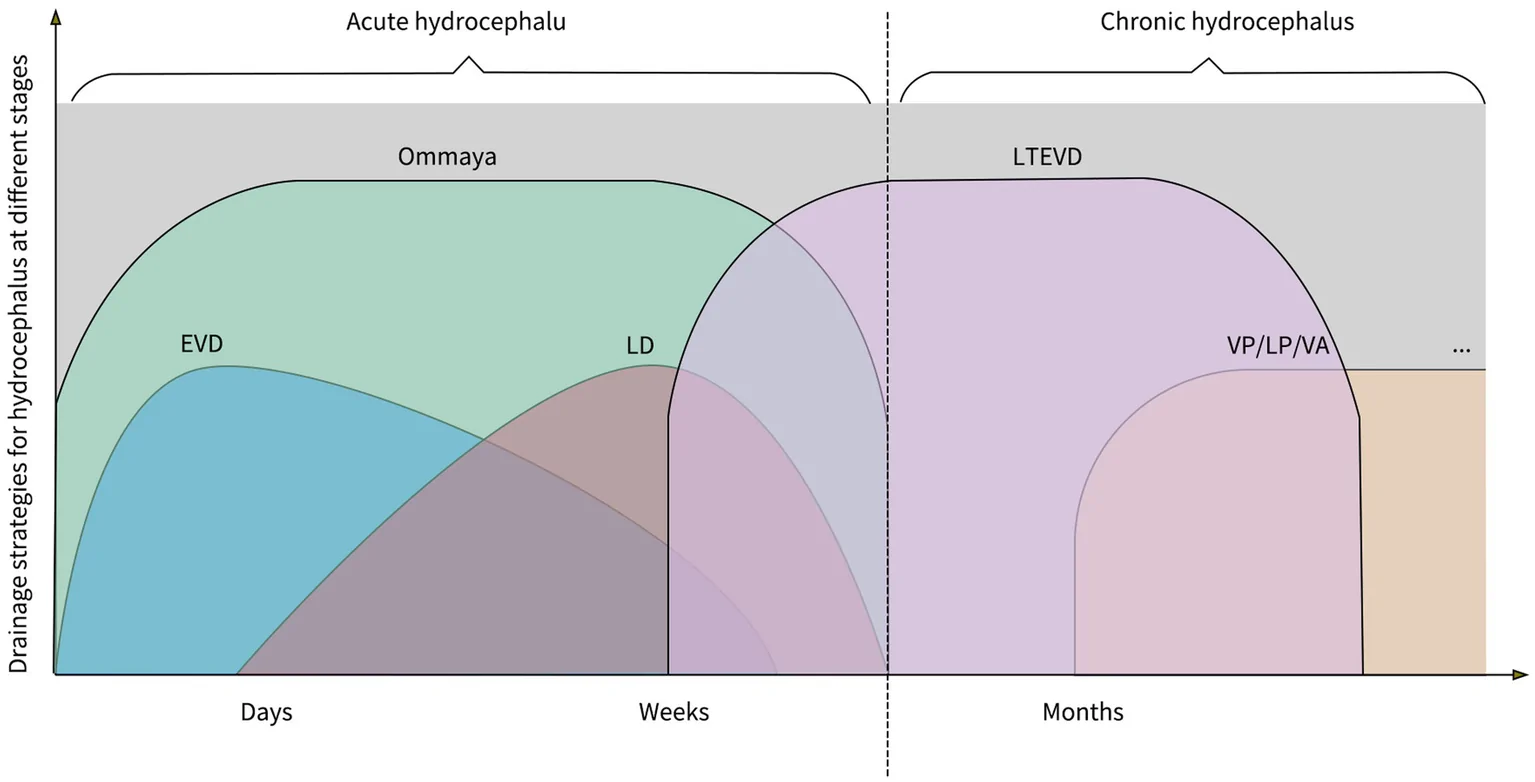
Cerebrospinal fluid drainage methods based on different disease courses.

For chronic hydrocephalus, options include Endoscopic Third Ventriculostomy (ETV) or permanent shunts (VP/VA/LP). If shunt infection occurs, administer antibiotics first—restart the shunt if controlled, or remove it and use implanted Ommaya (for drug delivery) and LD (for CSF drainage) if uncontrolled.

Traditional VP shunts and EVD, though classic, often fail to fully meet clinical needs: e.g., EVD patients with persistently high CSF red blood cell/protein levels cannot switch to internal drainage, or post-shunt removal (due to infection) with ongoing high intracranial pressure. Clinically, Ommaya EVD (combined with EVD), LD, and LTEVD serve as critical supplements. Neurosurgeons should comprehensively use these CSF drainage methods based on individual patient conditions to significantly improve hydrocephalus prognosis.

## Conclusion

The choice of hydrocephalus treatment depends on the acuity, underlying cause, and patient-specific factors. EVD and LD are preferred for acute cases requiring temporary drainage, while VP, LP, and VA shunts provide permanent solutions for chronic hydrocephalus. Ommaya reservoir EVD, LD and LTEVD offer alternatives for long-term temporary drainage needs. Understanding the indications, contraindications, and potential complications of each method is crucial for optimizing patient outcomes.
